# Piezoresistive Hydrogel-Based Sensors for the Detection of Ammonia †

**DOI:** 10.3390/s19040971

**Published:** 2019-02-25

**Authors:** Jan Erfkamp, Margarita Guenther, Gerald Gerlach

**Affiliations:** Solid-State Electronics Laboratory, Technische Universität Dresden, 01069 Dresden, Germany; mguenthe@mail.zih.tu-dresden.de (M.G.); gerald.gerlach@tu-dresden.de (G.G.)

**Keywords:** stimuli-responsive hydrogel, ammonia, pH value, alkaline pH conditions, chemical sensor, piezoresistive pressure sensor, hydrogel-based sensor

## Abstract

Ammonia is an essential key compound in the chemical industry. However, excessively high ammonia concentrations can be harmful to the environment. Sensors for the detection of ammonia are therefore particularly important for environmental analysis. In this article, a novel hydrogel-based piezoresistive ammonia sensor is presented. In aqueous solution, ammonia reacts as a base. This alkaline pH change can be detected with stimuli-sensitive hydrogels. For such an application, highly sensitive hydrogels in the alkaline range with sufficient mechanical stability for the sensor application has to be developed. These conditions are fulfilled by the presented hydrogel system based on acrylic acid (AAc) and 2-(dimethylamino)ethyl methacrylate (DMAEMA). The hydrogel composition has a significant influence on the swelling behavior of the gel. Furthermore, the hydrogel swelling in ammonia solutions was tested and a detection limit in the range of 1 mmol/L ammonia depending on the buffer solution was determined. Ammonia-sensitive hydrogels can be used multiple times due to the repeatable swelling of the gel over several swelling cycles. To generate a measurable output voltage, the swelling pressure of ammonia-sensitive hydrogels were detected by using piezoresistive pressure sensors. All results of the free hydrogel swelling were verified in the sensor application. This low-cost ammonia sensor with a high sensitivity could be interesting for industrial chemical and biotechnological applications.

## 1. Introduction

Ammonia (NH_3_) is one of the most important inorganic compounds in chemical manufacturing. In 2011, 140 megatons of ammonia were produced worldwide based on the Haber–Bosch process [1]. Ammonia is one of the key compounds for the production of fertilizers, pharmaceuticals, dyes or explosive materials [2]. However, excessively high ammonia concentrations can be a serious health threat [3]. Therefore, it is necessary to monitor ammonia as part of the ambient conditions in environmental analytics [4] or to measure the atmospheric pollution from automobiles [5]. Furthermore, ammonia can be used as an indicator for diseases like stomach ulcers due to bacterial infections [6] or a disturbed urea balance due to a kidney disorder [7]. According to the German Drinking Water Regulations from 2001, the limit value of dissolved ammonium ions in drinking water is 0.5 mg/L [8]. Depending on the application, it is necessary to detect ammonia in different concentration ranges [3]. 

In aqueous solutions, ammonia reacts as a base (pK_B_ = 4.75) to ammonium and hydroxide ions according to the following reaction equation [9]:

NH_3_ + H_2_O → NH_4_^+^ + OH^−^

The result is a change in pH value, which can be detected via pH-responsive hydrogels. Hydrogels are three-dimensional polymer networks which can absorb a huge amount of water. Due to the chemical modifications achieved by using different monomers, these gels have various swelling properties depending on different stimuli like salt concentrations, temperature or pH [10]. These gels can be combined with piezoresistive pressure sensor chips, which transform the swelling pressure into a measurable output voltage [11]. By the simple modification of this sensor set-up with different stimuli-responsive hydrogels, new types of sensors for a wide range of applications can be quickly and easily developed. For example, hydrogel-based piezoresistive sensors have been used so far for the detection of the pH value [12], temperature [13], biomolecules like glucose [14] or organic solvents like ethanol [15]. However, each hydrogel system must be adjusted to its specific sensor application. For the detection of ammonia, a hydrogel system which is highly sensitive in the alkaline pH range and has a sufficient mechanical stability for the usage in the pressure sensor has to be synthesized. In this work, we present a pH-sensitive hydrogel based on acrylic acid (AAc) [16,17,18] and 2-(dimethylamino)ethyl methacrylate (DMAEMA) [19,20,21], which fulfills all conditions for the detection of ammonia in a piezoresistive pressure sensor.

## 2. Materials and Methods

### 2.1. Synthesis of pH-Sensitive Poly Acrylic Acid-co-Dimethylaminoethyl Methacrylate Hydrogels

**Figure 1:**
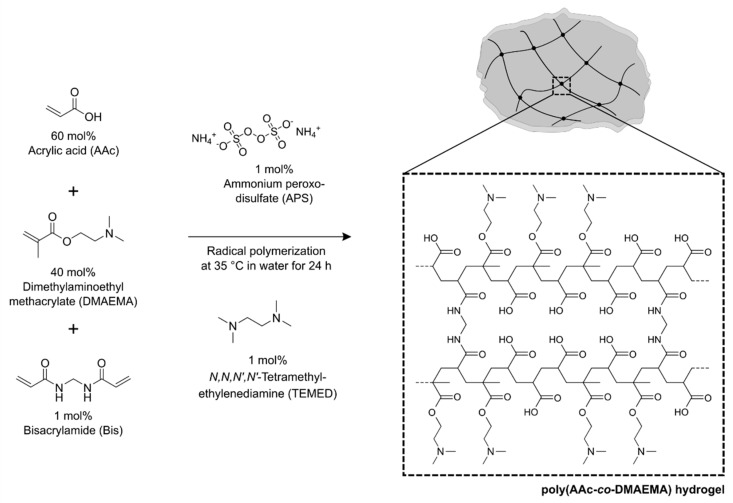
Synthesis of poly acrylic acid-*co*-dimethylaminoethyl methacrylate hydrogels.

For the detection of ammonia, a pH-sensitive hydrogel based on acrylic acid (AAc) and dimethylaminoethyl methacrylate (DMAEMA) was synthesized. The pregel solutions with different contents of AAc (x mol%, Sigma Aldrich, St. Louis, MO, USA) and DMAEMA (100-x mol%, Sigma Aldrich) were combined with 1 mol% of the crosslinker bisacrylamide (Bis, Carl Roth, Karlsruhe, Germany) and were dissolved in distilled water. In all cases, the total concentration of AAc and DMAEMA was 1.6 mol/L [22] (equivalent to 100 mol%). After degassing the pregel solution with nitrogen for 5 min, the polymerization was initiated with 1 mol% ammonium peroxodisulfate (APS, Sigma Aldrich) and 1 mol% *N*,*N*,*N*′,*N*′-tetramethyl-ethylenediamine (TEMED, Carl Roth). The total volume of a synthesis approach was 600 µL. As an example, for the synthesis of a poly(AAc-*co*-DMAEMA) hydrogel (60 mol% AAc and 40 mol% DMAEMA, 600 µL) will be used 576 µmol (39.5 µL) AAc, 384 µmol (64.7 µL) DMAEMA, 9.6 µmol (1.5 mg) Bis, 9.6 µmol (2.2 mg) APS and 9.6 µmol (1.5 µL) TEMED. The pregel solution was filled in a vial shell (1 mL, VWR, Radnor, PA, USA) and polymerized overnight in an oven at 35°C. After polymerization, vial shells were carefully smashed without damaging the hydrogel. One tablet of a phosphate-buffered saline (PBS, pH 7.4, *I* = 0.15 mol/L, Sigma Aldrich) was dissolved in 200 mL distilled water. The gels were cut in small pieces with a thickness of about 5 mm and were washed in PBS buffer, pH 7.4, for 3 days to remove unreacted monomers. The synthesis of ammonia-sensitive poly(AAc-*co*-DMAEMA) hydrogels is shown in Figure 1. 

### 2.2. Swelling Studies at Different pH Values

After washing, the gels were conditioned fivefold by cyclic changes between PBS solutions with pH 7.4 and pH 10. The pH values were adjusted by a sodium hydroxide solution (0.1 mol/L) using a pH meter (FiveEasy Plus, Mettler-Toledo, Gießen, Germany). Subsequently, the gels were washed in PBS buffer, pH 7.4, and the initial mass *m*_0_ of the hydrogel was measured using a balance (Entris 224-1S, Sartorius, Goettingen, Germany). After incubating in a PBS buffer with a defined pH value for overall 24 h accompanied by changing the buffer five times within 10 h to wash the gel, the hydrogel mass *m* after swelling and the pH value of the solution were detected. In the following measurements, the gels were incubated in a new PBS buffer with another pH values. The swelling degree *S* was calculated as:

*S* = (*m* − *m*_0_)/*m*_0_ (1)

### 2.3. Swelling Studies at Different Ammonia Concentrations

Swelling studies were performed in PBS buffer and in an isotonic saline solution (NaCl, 9 g/L, *I* = 0.15 mol/L). After washing and conditioning, the initial mass *m*_0_ of the hydrogel and the pH value were measured in PBS or NaCl solution without NH_3_. Ammonium hydroxide (≈1 mol/L ammonia in water, Sigma Aldrich) was used to prepare a 1 mol/L ammonia stock solution in PBS and NaCl buffer. A solution with a defined NH_3_ concentration was prepared with the stock solution and the gels were incubated for 24 h. After determination of the hydrogel mass *m*, the pH value and the swelling degree *S* according to Equation (1), the hydrogels were placed in a solution with another ammonia concentration for 24 h.

### 2.4. Repeatability of Hydrogel Swelling in Ammonia Solutions

Repeatability in ammonia hydrogel swelling was studied in PBS buffer solutions after washing and conditioning the synthesized hydrogels. According to the measurement of the initial mass *m*_0_ of the gels in PBS buffer, the gels were placed in a PBS buffer containing 20 mmol/L ammonia for 24 h. The hydrogel mass *m* after swelling and the pH value of the solution were measured. Afterwards, the solutions were changed to a PBS buffer without ammonia. In this case, the PBS buffer solutions were changed five times every 1–2 h to ensure a complete pH change in the gel and in the solution. After in total 24 h in PBS buffer without ammonia, the hydrogel mass *m* and the pH value of the solution were determined. Afterwards, the gels were incubated again in a PBS buffer with 20 mmol/L ammonia for 24 h. The switching between PBS buffer solutions with and without ammonia was repeated several times. At the end of the experiment, the gels were left in a PBS buffer without ammonia for several days and the mass *m* of the hydrogels was determined daily. The swelling degree was calculated as described in Equation (1).

### 2.5. Swelling Kinetics in Ammonia Solutions

As before, the hydrogels were washed and were conditioning after the polymerization in PBS buffer. Now the initial mass *m*_0_ of the gels was measured and the samples were placed into a 20 mmol/L ammonia solution in PBS buffer until the degree of hydrogel swelling remained constant. Afterwards, the gels were put into a PBS buffer solution without ammonia until the swelling degree of the gel was constant. To make sure to have a complete change of the pH value, the PBS buffer were changed daily. In both cases, the mass *m* of the swollen hydrogel and the swelling degree *S* according to Equation (1) were determined at defined points in time.

### 2.6. Hydrogel-Based Piezoresistive Ammonia Sensor 

**Figure 2:**
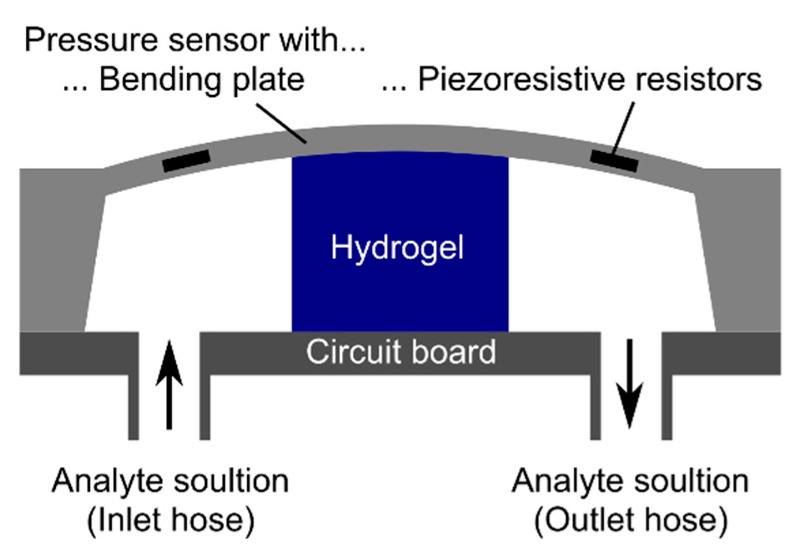
Schematic construction of a hydrogel-based piezoresistive pressure sensor. The figure was modified according to Erfkamp et al. [15].

For the synthesis of hydrogel pieces with a defined size, a plastic spacer with a thickness of 500 µm between two object slides was used during the polymerization. After washing and conditioning the hydrogel layers, circular hydrogel pieces with a diameter of 1.5 mm were punched out for sensor preparation. These hydrogel pieces were fixed in the center of a circuit board with cyanoacrylate. The pressure sensor chip (TDK Electronics, previously EPCOS, Munich, Germany, C41-Series, 5.0 mm × 5.0 mm × 0.4 mm in size) was put on the top of the gel and was fixed by using cyanoacrylate. After the wire bonding process of the pressure sensor chip to the circuit board, the connector cables were soldered to the read-out unit (Fluke 45 Multimeter, Glottertal, Germany). The outlet and inlet hoses for the ammonia solutions were fixed with cyanoacrylate and two-component epoxy adhesive [15]. The resulting hydrogel-based sensor is shown schematically in Figure 2.

The hydrogel in the sensor was washed over night in PBS buffer. Afterwards, the sensor was conditioned two times in a 20 mmol/L ammonia solution in PBS buffer for in total two days. In order to investigate the repeatability of hydrogel swelling, a PBS buffer solution with 20 mmol/L ammonia was pumped for 4 h. Subsequently, the solution was changed to a PBS buffer without ammonia for in total 20 h. This swelling cycle was repeated three times in total. For the determination of the ammonia sensitivity, ammonia solutions in a range from 1 mmol/L up to 20 mmol/L were prepared with PBS buffer. After the swelling of the hydrogel in PBS buffer without ammonia, the solutions were changed every 4 h to the next higher ammonia concentration. For pumping the solutions, a peristaltic pump (Reglo Digital MC-2/6, Ismatec as part of Cole-Parmer, Wertheim, Germany) with a constant flow rate of 0.2 mL/min was used in all experiments. The sensor was supplied with a voltage of 5 V (DIGI 35, Voltcraft as part of Conrad Electronic, Hirschau, Germany). The output voltage of the sensor was recorded every 10 s. All sensor measurements were performed at room temperature.

## 3. Results and Discussion

### 3.1. Swelling Studies at Different pH Values

**Figure 3:**
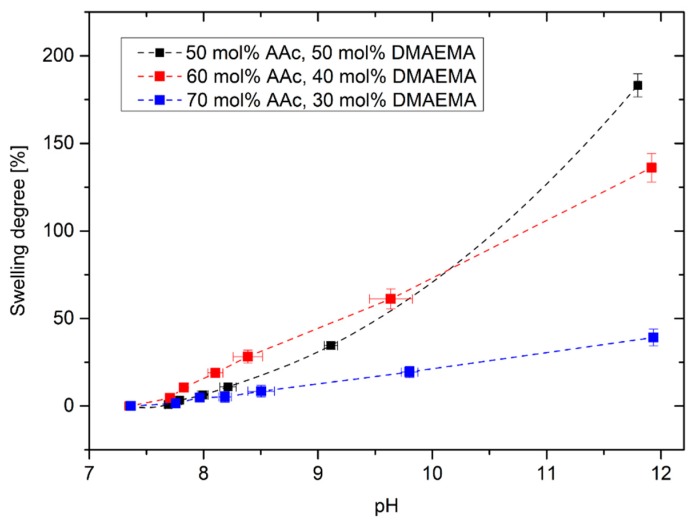
Swelling degree of poly(AAc-*co*-DMAEMA) hydrogels versus pH value for different monomer contents in PBS buffer.

At first, the swelling degree of poly(AAc-*co*-DMAEMA) hydrogels was investigated in dependence on the pH value for different monomer contents (Figure 3). Hydrogels having compositions of 50–70 mol% AAc and 30–50 mol% DMAEMA were successfully synthesized. For hydrogels with more than 70 mol% AAc, no complete polymerization was observed under the described conditions. Furthermore, hydrogels with more than 50 mol% DMAEMA could not be utilized under mechanical stress.

Hydrogels based on polyAAc swell under basic conditions because of the deprotonation of the carboxyl groups resulting in an increase of the electrostatic repulsion of the carboxylate anions in the gel [23]. In the literature, the pK_A_ value of polyAAc was determined in a range from 5.35 to 7.2 [24] depending on the actual dissociation degree of the polyAAc chain. As a result, the deprotonation should occur in a more acidic range. Due to the basic monomer DMAEMA (pk_B_ = 7.8 [10]), the operation range of the gel was shifted towards a more alkaline range [22]. Another effect that could influence the hydrogel swelling can be described with the *Donnan* theory. By increasing the pH value above pK_A_ value, the carboxyl group of acrylic acid is deprotonated. In order to neutralize the resulting charges, the moving counterions diffuse into the gel from the surrounding solutions. This leads to an increase of the ion concentration in the gel resulting in increasing osmotic pressure, which will be compensated by the diffusion of water molecules from the surrounding solution into the gel. As a result, the hydrogel swells with increasing pH value [25]. The higher the content of DMAEMA is, the higher becomes the swelling degree of the hydrogels. However, the polymer system with the highest swelling degree shows a non-linear swelling behavior. Hydrogels with 60 mol% AAc and 40 mol% DMAEMA were used in the following experiments because these gels showed a linear swelling behavior over a large pH range with a high sensitivity.

### 3.2. Swelling Studies at Different Ammonia Concentrations

**Figure 4:**
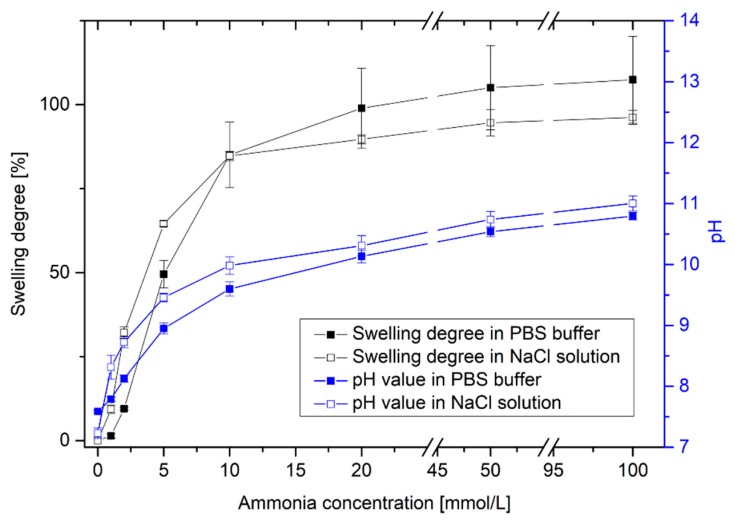
Swelling behavior of poly(AAc-*co*-DMAEMA) (60 mol% AAc, 40 mol% DMAEMA) hydrogels and resulting pH value for different ammonia concentrations in PBS buffer and isotonic saline solutions (NaCl).

After the pH-dependent swelling of poly(AAc-*co*-DMAEMA) hydrogels, the hydrogel swelling was determined as a function of the ammonia concentration in PBS buffer and in an isotonic saline solution (Figure 4).

With increasing ammonia concentration, the pH value of the solution increases significantly. This results in a swelling of the pH-sensitive hydrogel. Even small concentrations can be detected via swelling of the hydrogel. It was possible to detect small changes in pH value via hydrogel swelling. With this polymer system, it was also possible to detect small concentrations of ammonia. The hydrogel system is suitable for the detection of small amounts of ammonia in the range of 1 mmol/L to 20 mmol/L. This could be of particular interest for industrial chemical and biotechnological applications. 

Furthermore, a difference in the detection limit between PBS solution and isotonic saline solution could be observed. PBS buffer should keep the pH value in the physiological range as constant as possible. Due to the phosphate buffer, small changes in pH value can be compensated in the solution. With changing ammonia concentration from 0 mmol/L to 1 mmol/L NH_3_, the pH value increased only from pH 7.59 to pH 7.79 (∆pH = 0.20) in PBS buffer. However, in an isotonic saline solution, the pH value increased from pH 7.23 to pH 8.31 (∆pH = 1.08). As a result, a significant swelling of the hydrogels could be detected only in sodium chloride solution. The corresponding detection limit amounts to ca. 1 mmol/L NH_3_. In PBS buffer, a substantial swelling of the hydrogels was observed for concentrations of more than 2 mmol/L NH_3_. At higher ammonia concentrations, no relevant difference between the buffer solutions could be seen. 

### 3.3. Repeatability of Hydrogel Swelling in Ammonia Solutions

**Figure 5:**
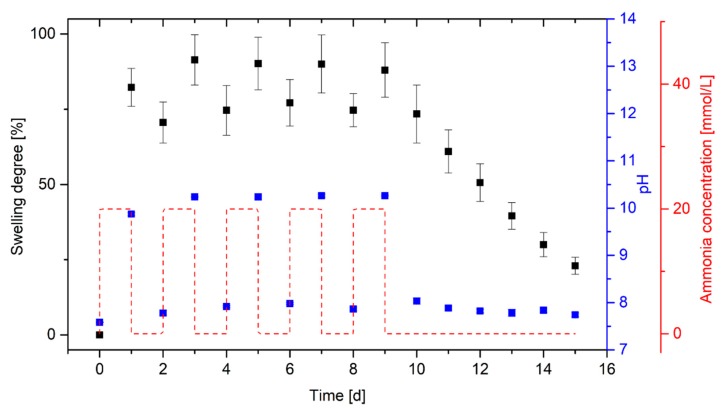
Repeated swelling of poly(AAc-*co*-DMAEMA) (60 mol% AAc, 40 mol% DMAEMA) hydrogels at two alternating ammonia concentrations (20 mmol/L and 0 mmol/L) in PBS buffer and resulting pH value of the solutions.

Repeatable swelling of the hydrogel is indispensable for a multiple use of the hydrogel-based sensors. For this purpose, the ammonia concentration in PBS buffer was varied daily and the degree of swelling of the hydrogels was determined (Figure 5).

Within 24 h, poly(AAc-*co*-DMAEMA) hydrogels swell significantly (*S* ≈ 82%) in the PBS buffer with 20 mmol/L ammonia due to the increase of the pH value (pH ≈ 9.9). After 24 h in PBS buffer without ammonia, a shrinking of the gels was observed. However, only a small change in the degree of swelling could be detected (Δ*S* ≈ 12%). This indicates that the hydrogels need more time for the shrinking process than for the swelling process. To verify this, the gels were stored for several days in PBS buffer without ammonia at the end of the reproducibility experiments. A steady decrease in the swelling degree was observed without a significant change in the pH value of the PBS solution. Asymmetric swelling behaviors were described for different kinds of stimuli-responsive hydrogels [26,27]. For example, Qu et al. also observed for chitosan-based hydrogels a longer response time for shrinking than for swelling in dependence on the pH value. During the swelling process, the functional groups of the gel surface will first become protonated. The resulting electrostatic repulsions lead to the swelling of the surface. Due to the swollen gel surface, the stimulus can diffuse more easily into the gel. During the shrinking process, the functional groups of the gel surface will be deprotonated at first. As a result, the gel surface deswells at first due to decreasing electrostatic repulsions. The shrunken gel surface hinders the diffusion of the stimulus into the gel compared to the swelling process and the gel requires more time for the shrinking process [28]. This effect can also influence the swelling behavior of the tested ammonia-responsive hydrogel. In addition, prioritized electrostatic interactions of ammonium and hydroxide ions with the charged groups of the hydrogel may be a possible explanation for the significantly longer dehydration compared to the hydrogel swelling. Due to the strong electrostatic forces between the mobile ions and the functional groups in the gel, the mobile ions diffuse out of the gel much more slowly. Furthermore, the hydrogel based on the acidic monomer acrylic acid and the basic monomer 2-(dimethylamino)ethyl methacrylate, is amphiphilic. Similar to pH buffer solutions, which are also amphiphilic, the hydrogel itself can compensate changes in pH value. As a result, the hydrogel needs a considerably longer time before the pH value in the gel has been adjusted to the pH value of the surrounding solution.

The hydrogels show almost equal swelling degrees (*S* ≈ 90%) after a total of five swelling cycles over 24 h in PBS solutions with 20 mmol/L ammonia. Only the first measurement (*S* ≈ 82%) deviates slightly from the further measurements. Before the measurements, all hydrogels were conditioned five times. By conditioning the hydrogels after synthesis, polymer chains that are too short break and the polymer chains reorient themselves in the gel. The drift during the swelling process has to be avoided by the pre-swelling, whereby the measuring accuracy increases significantly [11,29]. In this case, the drift indicates that the hydrogel system might need more than five conditioning cycles. However, the drift is quite small and its value is within the range of the uncertainty of measurement. The experiment shows that the hydrogels swells and shrinks reproducible, however, the gels require a considerably longer time for shrinking than for swelling. 

### 3.4. Swelling Kinetics in Ammonia Solutions

**Figure 6:**
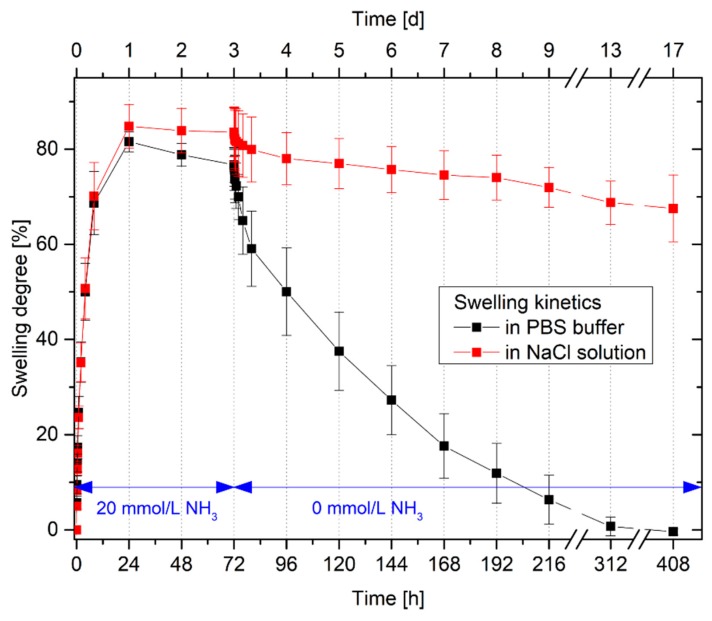
Swelling kinetics of poly(AAc-*co*-DMAEMA) (60 mol% AAc, 40 mol% DMAEMA) hydrogels in PBS buffer and in isotonic sodium chloride (NaCl) solution with 20 mmol/L ammonia and without ammonia, respectively.

Since the swelling kinetics of the hydrogel system seems to be very interesting and can give important information regarding the response behavior, it has been studied more in detail (Figure 6).

For the swelling process, the kinetics in PBS buffer and in an isotonic sodium chloride solution is nearly the same. In both solvent systems, the hydrogels are completely swollen after 24 h. Also the swelling degrees of the hydrogels do not differ significantly from each other in PBS buffer and the isotonic sodium chloride solution. The reduction of the degree of swelling in PBS buffers by a few percent lies within the limits of the uncertainty of measurement.

However, a considerable difference was found between the different solvent systems for the shrinking of the hydrogels. As already observed in the reproducibility tests, the hydrogels in PBS buffers require considerably more time for shrinking than for swelling. Possible explanations were discussed in Section 3.4. These results confirm the hypothesis assumed in the repeatability study of the hydrogel swelling. The hydrogels were completely deswollen after 14 days in PBS buffer without ammonia. Even in isotonic sodium chloride solutions, the hydrogels shrank, however the gels required more time than in comparison to the PBS buffer. After 14 days in an isotonic sodium chloride solution without ammonia, the degree of swelling decreases by just 16%. In this case, the pH value of the salt solution could have an influence on the swelling behavior. An isotonic salt solution is generally not neutral but more acidic because carbon dioxide in the atmosphere will be solved spontaneously in the solution. Carbon dioxide reacts in water as an acid [30]. In PBS buffer, the change in pH value can be compensated by the buffer system. However, in an isotonic sodium chloride solution, this is not possible. The acidic pH change can influence the swelling properties of the amphiphilic poly(AAc-*co*-DMAEMA) hydrogel [22]. For further investigations, a PBS buffer should therefore be used preferably. 

### 3.5. Hydrogel-Based Piezoresistive Ammonia Sensors

**Figure 7:**
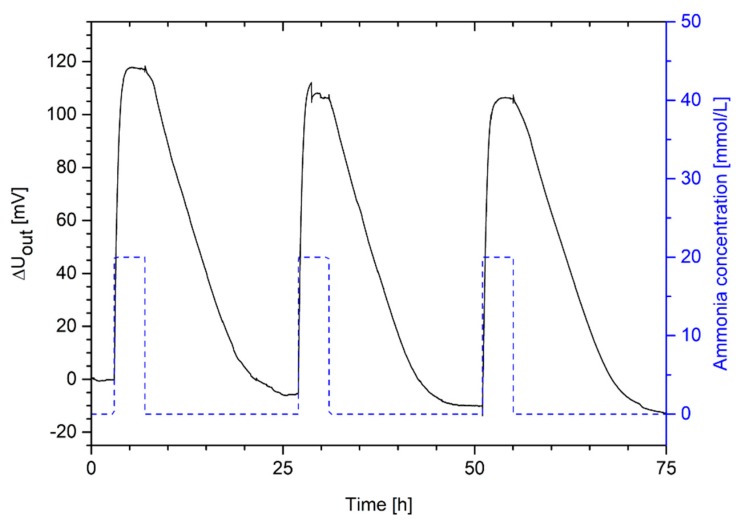
Change in output voltage of a hydrogel pressure sensor based on poly(AAc-*co*-DMAEMA) (60 mol% AAc, 40 mol% DMAEMA) for cyclic changes of ammonia concentrations in PBS buffer between 20 mmol/L and 0 mmol/L.

For the application in sensor technology, it is important to transform the swelling pressure of ammonia-sensitive hydrogels in a measurable output voltage. Piezoresistive pressure sensors chips, for example, can be used for this purpose. In a first step, the reproducibility of the hydrogel swelling in the sensor application was studied (Figure 7).

Hydrogel-based ammonia sensors can swell and deswell repeatedly over several swelling cycles. The change in the output voltage amounted in all cases to approximately 120 mV. This sensor measurement verifies the assumed reproducibility of the gel swelling discussed in Section 3.3. Like the swelling kinetics in Section 3.4, the hydrogel needs also a much shorter time for the swelling process (complete swelling within ca. 2 h) than for the shrinking of the hydrogel (complete deswelling within ca. 18 h). However, the response time of the hydrogel sensor is much lower than for free swelling. For sensor applications, much smaller hydrogel pieces were used compared to the free swelling experiments. The smaller a hydrogel is, the less time the stimulus needs to diffuse through the gel. In the case of spherical gels, the time constant for the swelling process decreases quadratically with the radius of the gel sample [29]. 

Furthermore, the hydrogel sensor showed a baseline drift during the measurement. Such a drift of the sensor signal has been observed for sensors based on different hydrogel systems [15,31,32]. The manual sensor preparation has a significant influence the sensor measurement [31]. Another effect could be the opposing forces of the bending plate that can compress the soft hydrogel after the swelling process [15]. As discussed in Section 3.3, an insufficient conditioning of the gels can also lead to a drift of the sensor signal [11,29]. Nonetheless, since the gels were pre-swollen for in total seven times (five times outside and twice inside of the sensor), the gels should be sufficiently conditioned. Therefore, this effect should influence the sensor measurement only marginally.

**Figure 8:**
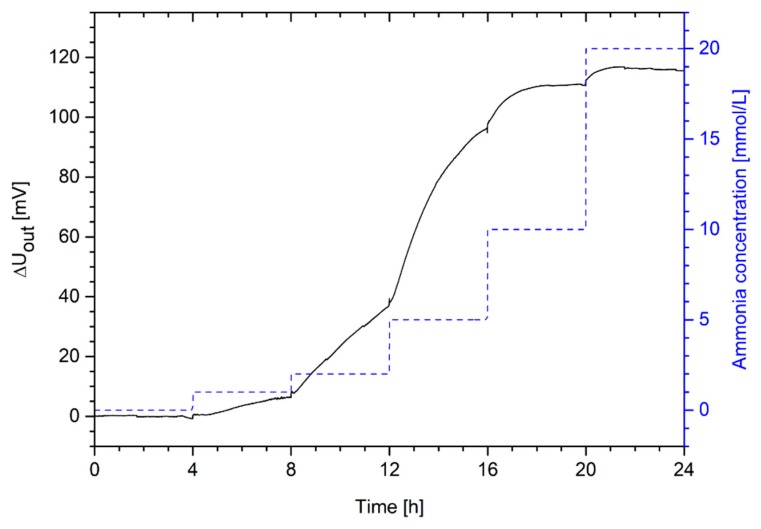
Change in output voltage of a piezoresistive ammonia sensor based on poly(AAc-*co*-DMAEMA) (60 mol% AAc, 40 mol% DMAEMA) hydrogels in a range from 0 mmol/L to 20 mmol/L ammonia in PBS buffer.

As known from the swelling studies in Section 3.2, the hydrogel system swells in solutions with ammonia concentrations in a range from 1 mmol/L to 20 mmol/L. For this reason, the sensitivity of the ammonia sensor was tested in that concentration range (Figure 8).

The hydrogel-based sensor is highly sensitive to ammonia concentrations. These results also confirm the hydrogel swelling results from Section 3.3. Even small concentrations of 1 mmol/L can be detected easily. The detection limit is thus below 1 mmol/L ammonia. Because of the signal repeatability, the low detection limit and its high sensitivity, this sensor might be especially interesting for industrial chemical and biotechnological applications.

## 4. Prospects for Commercial Sensing Applications

For the application of this sensor concept several decisive factors have to be considered. Although this principle can only be used for the detection of ammonia in aqueous solutions, this sensor might be applied in industrial chemical and biotechnological applications. For example, ammonia is used as refrigerant in cold storage facilities or indoor ice rinks. For monitoring the leakage of water-filled system parts and tubes, the company JUMO GmbH & Co. KG (Fulda, Germany) manufactures an ammonia sensor (Type 201040) for aqueous solutions. These sensor can detect dissolved ammonia in the ppm range [33]. According the German laws, the maximum occupational limit value is 40 ppm [34]. The detection limit of the hydrogel-based sensor is ca. 1 mmol/L, which equals about 18 ppm ammonia (1 ppm ≈ 1 mg/L [33] ≈ 55 μmol/L ammonia). Therefore, this in-line process-capable system can potentially be used for monitoring such ammonia limits. Furthermore, ammonia is often a by-product in biotechnological processes, e.g., in continuous mammalian cell lines for the production of pharmaceuticals. However, an excessively high ammonia concentration is toxic for the cells. For monitoring of ammonia in this process, the detection limit for this application has to be around 1 mmol/L [35]. This criterion is fulfilled by the presented hydrogel-based ammonia sensor. For biomedical applications, ammonia sensors with a detection limit in a range of up to ppb are necessary. For these applications, this sensor concept cannot be used due to the higher limit of detection.

Most application fields require sensors with a response time of a few minutes or seconds instead of hours or days [3]. At the moment, this is not possible with the current sensor set-up. However, response times for these sensors can be reduced significantly. As discussed in Section 3.5, the response time decreases with the size of the gel [29]. Smaller hydrogels in the sensor could further reduce response times, however, the hydrogel should be large enough to deform the bending plate of the pressure sensor. Additionally, the response times can also be reduced by using porous hydrogels. The stimuli can defuse much easier into the gel due to the porous structure, thus reducing the response time of the sensor. Based on a temperature-sensitive poly(*N*-isopropylacrylamide), Franke et al. could reduce the response time *t*_90_ from more than 300 min (for non-porous hydrogels) to ca. 10–20 s (for porous hydrogels) [13].

Furthermore, other cross-sensitivities can influence the measurement. This sensor principle is based on the indirect measurement of ammonia. As a result, the alkaline pH change due to the reaction of ammonia with water will be detected. Other bases can influence the sensor measurement. In addition, polyelectrolytic hydrogels like the tested polymer system swells not only in dependence on pH value but also in dependence on ionic strength. This phenomenon was shown with comparable polyelectrolytic hydrogels systems [36,37,38]. It can therefore be assumed that also this hydrogel system has a similar swelling behavior as a function to the ionic strength. For practical applications, it is necessary to separate the response to ionic strength from the response to pH. This can be achieved easily with an additional sensor using a neutral hydrogel [38]. For example, neutral polyacrylamide gels swell in dependence on the ionic strength [39] due to the change in osmotic pressure. However, these gels do not swell over a large pH range [40]. This separation should allow a reliable detection of the ammonia concentration.

## 5. Conclusions

Hydrogels have great application potential in sensing technology. Due to chemical modifications, new kinds of stimuli-sensitive hydrogels can be synthesized suitable for the measurement of the concentration of particular chemical species. The resulting swelling pressure can be sensed with piezoresistive pressure sensors. In this article, a new kind of ammonia-sensitive hydrogel was presented. Since ammonia reacts in water as a base, the ammonia concentration can be determined by using pH-sensitive hydrogels based on acrylic acid and 2-(dimethylamino)ethyl methacrylate. It was shown that this hydrogel system swells with a high sensitivity depending on the ammonia concentration. The swelling of the gel can be reproduced several times, allowing multiple use of this type of sensor. Furthermore, we were capable of proving the working principle of hydrogel-based ammonia-sensitive pressure sensors and could show that the results of the free-swelling experiments agree well with the sensor measurements.
